# Strangulated diverticulum: a new acute complication of small bowel diverticulosis

**DOI:** 10.1093/jscr/rjad253

**Published:** 2023-05-15

**Authors:** Renata Pajtak, Abdullah Ramadan, Paul Strauss

**Affiliations:** Department of General Surgery, Central Gippsland Health, 155 Guthridge Parade Sale, Melbourne, VIC 3850, Australia; Department of General Surgery, Central Gippsland Health, 155 Guthridge Parade Sale, Melbourne, VIC 3850, Australia; Department of General Surgery, Central Gippsland Health, 155 Guthridge Parade Sale, Melbourne, VIC 3850, Australia

**Keywords:** strangulated, jejunal, diverticulum

## Abstract

Complicated jejunal diverticulosis is a difficult entity to diagnose, which can cause significant morbidity and mortality. We present the case of an 88-year-old female who presented with a unique complication of small bowel diverticulosis progressing to a strangulated diverticulum requiring emergency surgery. We present the case of an 88-year-old female who presented with abdominal pain associated with a new mass on a background of perforated diverticulitis and previous laparoscopic abdominal surgeries for division of adhesions. Due to high suspicion for the mass containing necrotic bowel, the patient was taken directly to theatre for an exploratory laparotomy and was found to have ischaemic small bowel secondary to a strangulated jejunal diverticulum. When evaluating the acute abdomen consideration should be given to the diagnosis of a strangulated jejunal diverticulum causing ischaemic small bowel, with a view to expedite to emergency surgery as the primary treatment.

## INTRODUCTION

Diverticulosis of the small bowel is a rare entity occurring in 0.3–1.3% of patients [1]. It can result in acute complications including diverticulitis, perforation, intestinal bleeding and obstruction [2]. Complicated jejunal diverticulosis is a difficult entity to diagnose, which can cause significant morbidity and mortality. We present the case of an 88-year-old female who presented with a unique complication of small bowel diverticulosis progressing to a strangulated diverticulum requiring emergency surgical intervention.

## CASE REPORT

An 88-year-old female was admitted to the emergency department with a 6-hour history of lower abdominal pain and distension, vomiting and minimal oral intake. Her medical history was significant for syndrome of inappropriate antidiuretic hormone secretion, osteoporosis, hypertension, transient ischaemic attacks, gastroesophageal reflux disease, cholecystectomy, perforated diverticulitis and two previous laparoscopic abdominal surgeries for division of adhesions.

The patient reported that the pain started on the left side of the abdomen and was associated with a new mass. She also described associated symptoms of nausea and vomiting but denied any other symptoms including constipation and poor gas elimination. The patient reported having an abdominal wall hernia in the same region for the preceding few months, but she was yet to have this investigated.

On admission, the patient had a pulse of 80 bpm, blood pressure of 120/70 mmHg and was afebrile. Physical examination revealed abdominal distension, hyperactive bowel sounds on auscultation and tympanic sounds on percussion. There appeared to be a mass on the left side of the abdomen that did not cross the midline; and appeared to have a central area of fluctuance with surrounding cellulitis. Blood tests showed leukocytosis 15.4 × 10^9^/L, C-reactive protein 154 mg/L and a mild liver function test derangement. All other bloods were within normal parameters.

**Figure 1:**
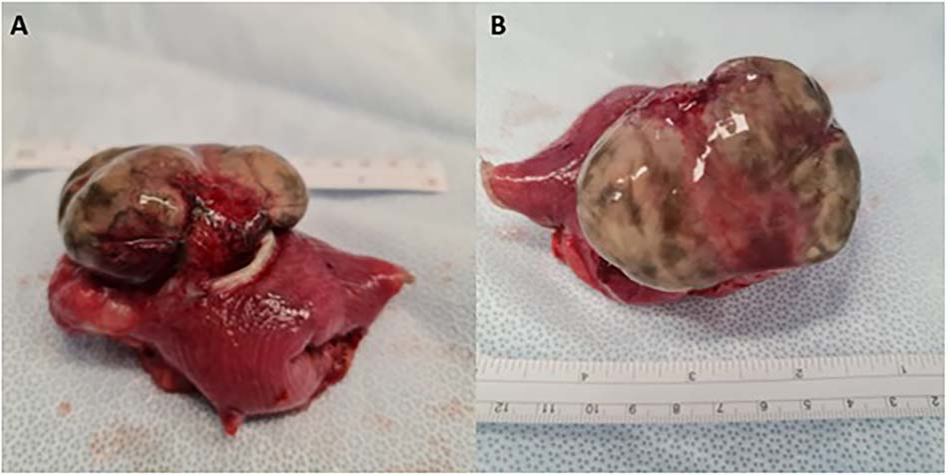
Small bowel diverticulum located in the mid-jejunum. (**A**) Transverse view (**B**) Superior view. Segment of small bowel 70 mm in length with a maximum internal circumference of 60 mm. There is attached mesentery up to 40 mm thick. There is an outpouching in the serosal surface measuring 85 × 50 × 25 mm. The overlying serosa appeared thinned and was stained green and there was surrounding hemorrhage at the base of the defect within the wall.

Due to the high suspicion for the mass containing necrotic bowel, the patient was taken directly to theatre for an exploratory laparotomy. The abdominal cavity was explored detecting a lower midline incisional hernia with strangulated, gangrenous small bowel diverticulum in the mid-jejunum (Fig. 1) and diverticulosis throughout the remainder of the small bowel. The hernial content was reduced. The section of bowel containing the strangulated diverticulum was resected and a side-to-side anastomosis performed using 3 × 60 mm blue gastrointestinal anastomosis (GIA) stapler after ligation and diathermy of the supplying region of mesentery. A region of necrotic omentum inside the hernia was also excised. The abdominal cavity was irrigated with 4 L of saline. A 10Fr blake drain was inserted into the hernial defect. Closure of the hernial defect and rectus sheath was achieved with 2 × polydioxanone suture (PDS) and monocryl 3-0 to skin. The patient had a good post operative recovery and was able to be discharged home.

### Position of figure

The histopathological study reported necrosis in keeping with ischaemia of a small bowel diverticula. Sectioning of the serosal outpouching (Fig. 1) revealed multiple diverticula. Microscopically there was evidence of transmural necrosis and suppurative inflammation, which was extending into adjacent tissue. Acute serosal inflammation was also present.

## DISCUSSION

The incidence of diverticulitis is higher in men (58%) then women (42%) [3]. Jejunal Diverticula are the most uncommon of all small bowel diverticula varying from an incidence of 0.2–1.3% in autopsy studies [4]. When affected, however, 75% of diverticula are located in the proximal jejunum, followed by the distal jejunum (20%) and then the ileum (5%) [5]. The higher incidence of diverticula in the jejunum compared to the ileum is attributed to the larger diameter of the penetrating jejunal arteries. Dysmotility or abnormalities within the myenteric plexus are also strongly corelated with the development of jejunal diverticulitis [6].

Diverticula themselves are herniations of the mucosae and submucosa through the muscular layer of the bowel wall. They often occur on the mesenteric border which distinguishes them from the congenital Meckel’s diverticulum [7]. The diagnosis of jejunal diverticulitis is often missed or delayed due to nonspecific symptoms of acute abdominal pain with symptoms of diarrhea being present in 58% of cases, chronic abdominal pain in 51% and bloating 44% [8]. The diagnosis of small bowel diverticular disease is typically made on computed tomography scan or via magnetic resonance imaging [9]. In our case the clinical acute abdomen signified the need for surgery and therefore pre-operative imaging was not performed.

Complications secondary to jejunal diverticulosis have been reported to occur in 46% of cases and include mechanical obstruction, intussusception, volvulus, perforation, peritonitis, bleeding and fistula formation [10, 11]. Given their higher rates of complication surgical management is often preferred to conservative management [12]. Furthermore, the literature supports that for perforated and obstructed jejunal diverticula causing generalized peritonitis, an expeditated laparotomy with segmental intestinal resection and primary anastomosis is indicated [13].

There has been one previous report of a strangulated jejunal diverticulum by Dangi *et al*. [10] in which it was found that an epigastric hernia contained strangulated jejunal diverticula which required resection and end-to-end jejunal anastomosis. However, we are the first to report small bowel ischaemia secondary to a strangulated diverticulum within an incisional hernia. The mechanism is thought to be due to the formation of adhesional bands at the base of the diverticula formed after repeated diverticulitis causing strangulation of the intestine [4].

We are the first to report a strangulated jejunal diverticulum causing ischaemic small bowel in a patient. We believe this diagnosis should be considered in the future as a differential for presentations of the acute abdomen, with consideration of expedition to surgery being key in safe and efficient management of the patient.

## Data Availability

Data available within the article or its supplementary materials.
